# The relationship between HIV-related stigma and HIV self-management among men who have sex with men: The chain mediating role of social support and self-efficacy

**DOI:** 10.3389/fpsyg.2022.1094575

**Published:** 2022-12-19

**Authors:** Yan Tao, Xueling Xiao, Jun Ma, Honghong Wang

**Affiliations:** Xiangya Nursing School, Central South University, Changsha, Hunan, China

**Keywords:** HIV, men who have sex with men, HIV-related stigma, social support, self-efficacy, self-management

## Abstract

HIV infection becomes a manageable disease, and self-management is one of the key indicators of achieving optimal health outcomes. Men who have sex with men (MSM) living with HIV face many psychosocial challenges when managing HIV infection, such as sexual minority pressure and HIV-related stigma. Higher perceived HIV-related stigma had been related to low self-management. However, the mechanisms underlying the association between HIV-related stigma and HIV self-management are unclear. Two possible mediators include social support and self-efficacy. This study aimed to examine the relationship between HIV-related stigma and HIV self-management among MSM living with HIV and to explore the single mediating effect of social support and self-efficacy and the chain mediating effect of these two variables on this relationship in China. Convenience sampling was used to recruit participants from the Center for Disease Control (CDC) in Changsha City, Hunan province, China. A total of 459 MSM living with HIV completed questionnaires regarding sociodemographic and disease-related information, HIV-related stigma, social support, self-efficacy, and HIV self-management. Descriptive statistics analysis, one-way ANOVA, independent *t*-tests, Pearson’s bivariate correlation, and multiple regression were conducted using the SPSS v24.0. Process macro in SPSS was used to analyze the single and chain mediating effect among variables. Our findings showed that the indirect and total effect of HIV-related stigma on HIV self-management was significant, while the direct effect was not statistically significant. Social support and self-efficacy mediated the relationship between HIV-related stigma and HIV self-management, respectively. Moreover, the chain mediating model confirmed that the association between HIV-related stigma and HIV self-management was mediated by social support and self-efficacy sequentially. Future interventions focusing on improving HIV self-management among MSM living with HIV should consider a multi-faced approach.

## Introduction

With the introduction and widespread use of antiretroviral therapy (ART) to decrease viral loads and prevent the HIV epidemic, HIV infection has become a manageable chronic disease (WHO, 2016; Bassett et al., 2019). However, the rate of new HIV infection is rising rapidly among men who have sex with men (MSM), about 28 times higher than that in general adult men (UNAIDS, 2020). In China, the average HIV infection rate among MSM is 9.2% (Zhuang et al., 2018), far exceeding the standard warning line of 5% for key populations set in international guidelines (UNAIDS, 2021). MSM continues to present the majority of HIV prevalence (Zhuang et al., 2018). Nowadays, the traditional medical model, which focuses on managing a specific disease and relies on the hospital or other health facilities, no longer meets the increased health needs of people living with HIV (PLWH) as the condition requires life-long treatment and PLWH often living with other non-AIDS defining chronic conditions (Zhang et al., 2019a; WHO, 2022a). The World Health Organization (WHO) suggested that self-management is the best practice for chronic care since individuals often require changes in daily lifestyle and day-to-day disease management (WHO, 2002). Self-management refers to the ability of patients to work with caregivers, community, and professionals to manage the physical, psychosocial, and lifestyle consequences of their chronic condition (Lorig and Holman, 2003). Recent studies reported that good self-management could, directly and indirectly, reduce susceptibility to worsening HIV, individual care expenditures, and the burden on healthcare system resources (Zhang et al., 2019b; WHO, 2022b). Given the complexity of the tasks of HIV self-management to achieve optimal outcomes, HIV self-management remains challenging and is influenced by individual, social and physical factors as mentioned in the contextual dimension of the Individual and Family Management Framework (IFSMT; Ryan and Sawin, 2009). Among those influencing factors, HIV-related stigma has been widely proven to be one of the biggest challenges and key predictors of HIV self-management (Sayles et al., 2009; Babel et al., 2021). In addition, according to the IFSMT, the process of enhancing social support and self-efficacy is necessary for chronic conditions management, which may influence HIV self-management behaviors and could be influenced by risk and protective factors in the contextual dimension. However, no studies have focused on the mechanisms underlying the relationship between HIV-related stigma and HIV self-management. Hence, the current study aims to investigate the mediating mechanisms by understanding the factors that mediate the relationship between HIV-related stigma and HIV self-management and provide a reference for improving HIV self-management.

### The association between HIV-related stigma and HIV self-management

Since the beginning of the HIV epidemic, PLWH have experienced various forms of HIV-related stigma, especially among marginalized groups such as MSM. There is a growing body of conceptual frameworks indicating that perceived HIV-related stigma decreases MSM living with HIV in getting appropriate treatment (Meyer, 2003; Stangl et al., 2019; Batchelder et al., 2021). Previous empirical studies also proved that stigma was associated with short-term behavior changes, such as lower medication adherence and more sexual risk behaviors, and the impact of behaviors on long-range adverse health outcomes, like depression and poor quality of life (Wu et al., 2015; Yigit et al., 2020; MacLean and Wetherall, 2021; van der Kooij et al., 2021). However, Zeng et al. (2020) revealed that anticipated stigma among PLWH did not directly and significantly predict self-management behaviors, including the level of treatment adherence. Due to the inconsistent results of the study, stigma may be indirectly associated with HIV self-management through other mediators, which has been ignored in most research. Furthermore, previous studies only explored the association between stigma and HIV self-management among general PLWH (Webel et al., 2012b; Wang H. et al., 2019; Wang H. H. et al., 2019; Areri et al., 2020a), and limited evidence from MSM living with HIV. Therefore, it is critical to explore potential pathways underlying HIV-related stigma and HIV self-management among MSM living with HIV.

### Mediating effect of social support

Social support might be a potential mediator in explaining how HIV-related stigma may lead to poor self-management. However, few studies have investigated whether social support operates as a mediator for the negative effects of HIV-related stigma on HIV self-management. According to the IFSMT (Ryan and Sawin, 2009), improving social support (e.g., peers and family support and use of communications) is necessary for chronic conditions management. Home and community environments have a critical influence on the intention of patients to obtain support. However, stigma toward PLWH often occurs in families, communities, and healthcare settings (Marshall et al., 2017; Nyblade et al., 2019). Numerous studies have shown that patients who experienced and anticipated stigma are less willing to disclose their HIV status to others for fear of rejection and exclusion, which motivates them to avoid the social situation (Parker and Aggleton, 2003; Hedge et al., 2021; Ndione et al., 2022). Meanwhile, previous studies have confirmed that a high level of social support was linked with optimal self-management behaviors (Aguilera-Mijares et al., 2022). For example, Du et al. (2018) indicated that a better social support MSM living with HIV obtained was associated with higher condom use intentions. Despite growing evidence of the positive impact of social support, the role of social support in the relationship between HIV-related stigma and HIV self-management remains unclear. Based on existing evidence (Ryan and Sawin, 2009; Du et al., 2018), the present study hypothesizes that the impact of HIV-related stigma on HIV self-management will be mediated by social support.

### Mediating effect of self-efficacy

Self-efficacy might be another potential mediator explaining the pathway from HIV-related stigma to HIV self-management. On the one hand, HIV-related stigma and self-efficacy are closely related. Zhou et al. (2020) conducted a study among 2,987 PLWH and found that self-efficacy tends to be lower among those who reported a higher HIV-related stigma. On the other hand, the link between self-efficacy and HIV self-management has been well established. Empirical and conceptual studies asserted that patients with higher self-efficacy are more likely to engage in effective healthcare strategies and demonstrate better persistence and effort to achieve successful viral suppression and obtain a better quality of life (Bandura, 1989; Johnson et al., 2007; Ryan and Sawin, 2009; Khumsaen and Stephenson, 2017). A study in China (Zhang et al., 2016) indicated that adherence self-efficacy was positively associated with HIV self-management, like medication adherence, among PLWH. Although the mediation role of self-efficacy between HIV-related stigma and HIV self-management has not been investigated, it has been found to be a mediator between stigma and medication adherence (Seghatol-Eslami et al., 2017), indicating that self-efficacy could buffer the negative impact of HIV stigma on behaviors in PLWH. Considering the association of self-efficacy with HIV self-management and stigma (Zhang et al., 2016; Zhou et al., 2020), self-efficacy may alleviate the negative effect of HIV-related stigma and further improve HIV self-management.

### The chain mediating effect of social support and self-efficacy

As we hypothesized, social support and self-efficacy mediate the relationship between HIV-related stigma and HIV self-management. However, when they were both considered to be mediators, what was the relationship between social support and self-efficacy? Which one played a more critical moderating role? According to the IFSMT (Ryan and Sawin, 2009), social support and self-efficacy in the self-management process are interrelated, which means better social support is internally related to higher self-efficacy. Aligning with the IFSMT, Zakiei et al. (2022) suggested that social support from family, friends, and others could indirectly affect risky behaviors through self-efficacy. Many studies have proven that the more social support patients perceive, the higher the possibility that they believe in their abilities to master disease management tasks (Levitt et al., 2017; White et al., 2020). Given the theory and empirical evidence (Ryan and Sawin, 2009; Zakiei et al., 2022), the present study hypothesizes that social support and self-efficacy will serially mediate the relationship between HIV-related stigma and HIV self-management.

**Figure 1:**
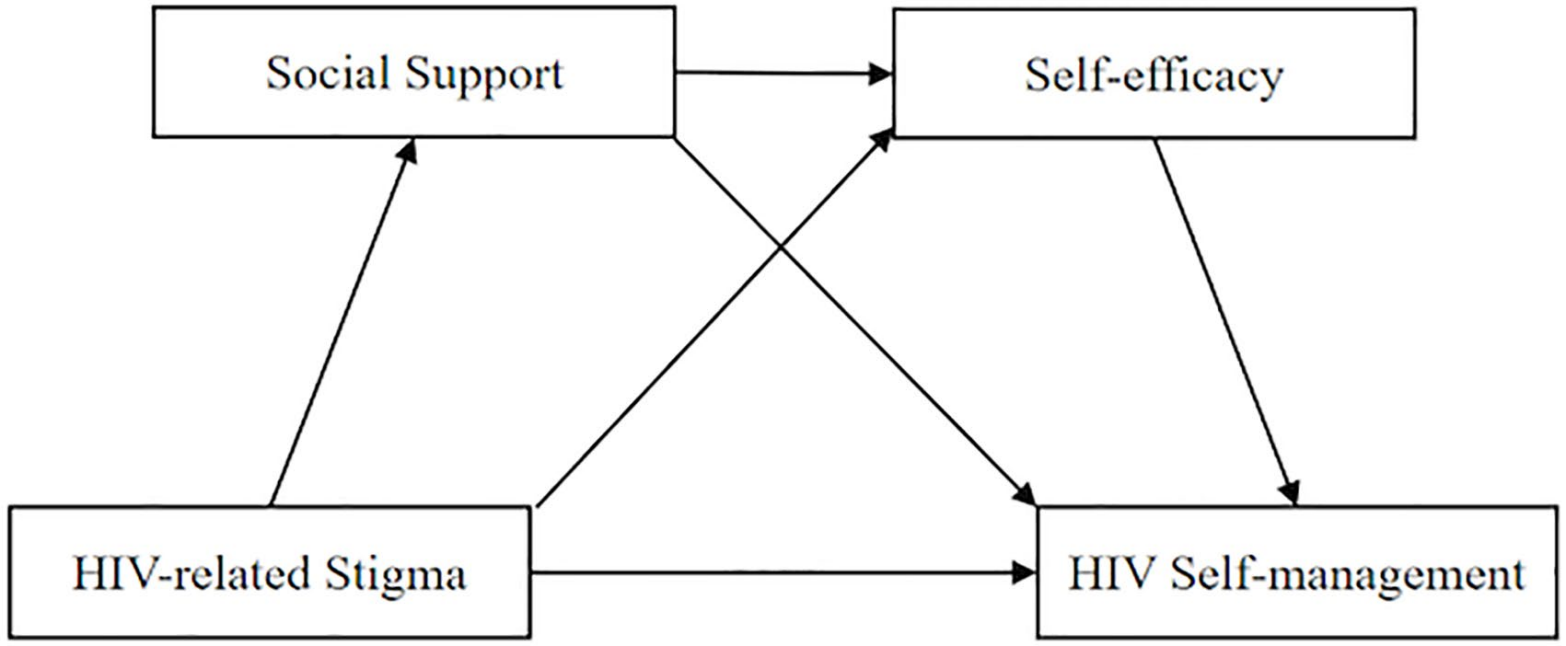
Hypothesized relationship among HIV-related stigma, social support, self-efficacy, and HIV self-management.

In summary, although the relationships among the variables of stigma, social support, self-efficacy, and health behaviors have been examined separately, the role of social support and self-efficacy in the impact of HIV-related stigma on HIV self-management among MSM living with HIV has not yet been thoroughly tested to date. Hence, we used IFSMT as the framework to test the relationship between HIV-related stigma and HIV self-management and the mechanical roles of social support and self-efficacy among MSM living with HIV. Based on previous research, we proposed three main hypothesized models (Figure 1):

*Hypothesis 1:* HIV-related stigma might have a negatively predictive effect on HIV self-management.

*Hypothesis 2:* Social support would act as a mediator between HIV-related stigma and HIV self-management.

*Hypothesis 3:* Self-efficacy would act as a mediator between HIV-related stigma and HIV self-management.

*Hypothesis 4:* Social support and self-efficacy would jointly act as a chain mediating role in the relationship between HIV-related stigma and HIV self-management.

## Materials and methods

### Participants

This cross-sectional study was conducted from October 2021 to January 2022. By convenience sampling, all participants were recruited from the Center for Disease Control (CDC) in Changsha City, Human Province, China. A set of self-reported questionnaires were used to collect data. A total of 473 questionnaires were distributed, of which 459 questionnaires were valid. Fourteen questionnaires (3%) were excluded due to participants dropping out in the middle of the process, resulting in an efficiency of 97.0%. The inclusion criteria were: (a) men meeting the Chinese Ministry of Health’s diagnostic criteria for HIV; (b) self-reported ever having sex with another man within the last 6 months; (c) 18 years or older; (d) provided informed consent; and (e) volunteered freely for the study. We asked a couple of questions to assess participants’ eligibility and excluded those with a severe mental disorder and linguistic or cognitive impairment.

### Procedures

The Ethics Committee approved all the study procedures of XXX. Participants were recruited by convenience sampling when they came to the AIDS department of CDC to get HIV health counseling or services. Four trained CDC staff introduced our project to the potential participants, and if they were willing to join this study, they were contacted and invited by the trained investigators. Before the investigation, the investigators explained the study’s purpose, content, significance, and potential risks to the participants. All participants provided written informed consent and voluntarily participated in the study. Subsequently, the investigators distributed paper-based questionnaires and explained the filling requirements to the respondents. All questionnaires were self-reported, and participants completed them independently in a private and quiet room to ensure privacy. A face-to-face interview was used for patients with limited reading ability to help them complete questionnaires.

### Measures

#### Demographic and disease-related variables

The research team designed the demographic survey to gather participants’ demographic information including age, educational level, residence, marital status, and monthly household income. HIV-related information, including HIV infection route, years of HIV diagnosis, comorbidities, and drug side effects, was also collected.

#### HIV self-management

The HIV Self-management Scale (HIVSMS) developed by Webel et al. (2012a), as revised by Han et al. (2019), was adopted to measure HIV self-management of PLWH. The scale consists of 20 items across three dimensions: daily physical health practices, activating HIV support groups, and living with chronic HIV conditions. The HIVSMS was scored on a four-point Likert scale (0 = not applicable, 1 = none of the time, 2 = some of the time, and 3 = all the time), with a higher score indicating a better status of self-management. The Chinese version of HIVSMS has good reliability, which has been applied and verified in Chinese PLWH (Wang H. et al., 2019). The Cronbach’s alphas of the subscales in this study are reported from 0.71 to 0.86.

#### HIV-related stigma

HIV-related stigma was assessed using a brief version of Berger’s Stigma Scale (HSS), which was developed by Berger et al. (2001) and shortened by Wright et al. (2007). The scale includes 10 items that inflect personalized stigma, disclosure concerns, negative self-image, and public attitudes. Each item’s statements were rated on a four-point Likert response ranging from 1 (strongly disagree) to 4 (strongly agree), giving composite scores ranging from 10 to 40. The higher the total score indicated the higher level of HIV-related stigma. In the current study, the Cronbach’s alpha of the entire scale was 0.87.

#### Social support

Social support was assessed using the Social Support Rating Scale (SSRS), initially designed in Chinese by Xiao (1994). The scale contains three subscales and 10 items, including subjective support, objective support, and the utilization of support. The composite scale score was the total of all items’ scores, and possible scores ranged from 12 to 66. The higher score a participant obtained, the better social support they had. The SSRS has been demonstrated to be a reliable and valid measure for assessing social support status in Chinese MSM living with HIV samples (Liu et al., 2017). In the study, the Cronbach’s alpha of the whole scale was 0.80.

#### Self-efficacy

Self-efficacy was measured using the Self-efficacy for Managing Chronic Disease Scale (SEMCDS) compiled by Lorig et al. (2001). The scale consists of six items and two subscales, symptom management self-efficacy and general self-efficacy. All items’ response options were measured on a 10-point Likert scale from 1 (no confidence at all) to 10 (extremely confident). Items were summed to get a total score ranging from 6 to 60, with higher scores indicative of higher levels of self-efficacy. The SEMCDS has been extensively validated with good psychometric properties among Chinese MSM living with HIV (Li et al., 2021). The overall Cronbach’s alpha in this study was reported to be 0.93.

### Data analysis

Descriptive statistics of sociodemographic and disease-related characteristics and variables of interest were reported. Frequency and percentage were used to describe categorical variables. Continuous variables such as scale total scores were reported on the mean (*M*) and standard deviation (*SD*). One-way ANOVA and independent *t*-tests were used to examine the differences in HIV self-management among sociodemographic characteristics and disease-related factors. Multiple linear regression analysis was conducted to further explore the associated factors if they had statistical significance in univariate analysis. Pearson correlation analyses were performed to examine whether there is a correlation between HIV stigma, social support, self-efficacy, and HIV self-management.

The direct and indirect effects of HIV-related stigma on HIV self-management were examined using bootstrap analyses with 5,000 bootstrap samples (Hayes, 2013). Adjusting for covariates that were significant in multivariate regression analysis, hypothesized single and chain mediation models were examined through the macro-program PROCESS 3.5 developed by Hayes (Preacher and Hayes, 2008; Hayes, 2013). Single mediation models of HIV-related stigma to HIV self-management through social support and self-efficacy were examined using PROCESS Model 1 (Preacher and Hayes, 2008). The chain mediation model was conducted to examine the path between two mediators in sequence and the indirect effects of each mediator independently using PROCESS Model 6 (Hayes, 2013). The mediating effect was significant if the 95% bias-corrected confidence interval did not include zero. A value of *p* of 0.05 (two-tailed) was considered statistical significance. All the data analyses and processing were completed using IBM SPSS v24.0 software.

## Results

### Common method biases tests

Due to a single source of data, Harman’s single-factor test was used to reduce the common method biases. The results revealed 13 factors with an eigenvalue greater than 1, and the total variation explained by the first factor was 16.73%, which was far lower than the critical value of 40%. Thus, there were no apparent common method biases in the data.

### Descriptive statistics

Table 1 presents participants’ sociodemographic and HIV-related characteristics and the corresponding distributions of HIV self-management scores. Overall, the mean age of the 459 participants was 30 years old (*SD* = 8.64, range: 18–76). The total HIV self-management score was 39.60. Monthly household income, infection route, comorbidities, and drug side effects were significantly associated with HIV self-management in univariate analyses. The multiple linear regression analyses included all the significant variables found in univariate analyses, that comorbidities and drug side effects were statistically significantly associated with HIV self-management, and were included in the single and chain mediation models as covariates. Participants with comorbidities or drug side effects reported a lower level of HIV self-management.

**Table 1:** Baseline characteristics and difference in the score of HIV self-management.

Variables	*N* (%)^a^	Self-management (*M* ± *SD*)	*t/F* ^b^	*p*
Total	459(100)	39.60 ± 8.31		
Age			0.421	0.656
≤30	225(49.0)	39.64 ± 8.22		
30–40	179(39.0)	39.28 ± 8.34		
>40	55(12.0)	40.45 ± 8.64		
Educational level			0.352	0.704
Senior high school or lower	78(17.0)	39.474 ± 8.13		
Junior college	146(31.8)	39.18 ± 8.53		
Undergraduate or higher	235(51.2)	39.90 ± 8.25		
Residence			0.393	0.531
Countryside	225(49.0)	39.35 ± 8.56		
City/town	234(51.0)	39.84 ± 8.07		
Marital status			0.828	0.363
Married	153(33.3)	40.10 ± 8.67		
Unmarried	306(66.7)	39.35 ± 8.12		
Monthly household income (Chinese yuan)			3.365	0.035
<10,000	184(40.1)	38.98 ± 8.61		
10,000–20,000	142(30.9)	38.94 ± 7.72		
≥20,000	133(29.0)	41.17 ± 8.33		
Infection route			5.558	0.011
Fixed partner	56(12.2)	42.64 ± 8.60		
Other partners	351(76.5)	39.07 ± 8.20		
Other infection routes	52(11.3)	39.60 ± 8.31		
Yeas since HIV diagnosis			0.846	0.358
<5	269(58.6)	39.35 ± 8.31		
≥5	190(41.4)	40.08 ± 8.29		
Having comorbidities			16.297	<0.001
No	388(84.5)	40.26 ± 8.12		
Yes	71(15.5)	36.00 ± 8.46		
Having drug side effects			9.346	0.002
No	208(45.3)	40.89 ± 8.61		
Yes	251(54.7)	38.53 ± 7.90		

a Numbers are unweighted, but percentages are weighted.

b *t*, *t* test; *F*, one way ANOVA.

### Bivariate correlations among main variables

As shown in Table 2, higher HIV-related stigma was correlated with lower social support (*r* = −0.338, *p* < 0.001), lower self-efficacy (*r* = −0.295, *p* < 0.001), and lower HIV self-management (*r* = −0.141, *p* < 0.01). Higher social support was correlated with higher self-efficacy (*r* = 0.294, *p* < 0.001) and better HIV self-management (*r* = 0.240, *p* < 0.001). Higher self-efficacy was correlated with better HIV self-management (*r* = 0.349, *p* < 0.001).

**Table 2:** Means, standard deviations, and correlations for study variables (*N* = 459).

		*M*	*SD*	1	2	3	4
1	HIV-related stigma	28.47	5.89	1			
2	Social support	30.69	7.55	−0.338^***^	1		
3	Self-efficacy	42.07	10.76	−0.295^***^	0.294^***^	1	
4	HIV self-management	39.60	8.31	−0.141^**^	0.240^***^	0.349^***^	1

^**^p < 0.01, ^***^p < 0.001.

### Single mediation model

**Figure 2:**
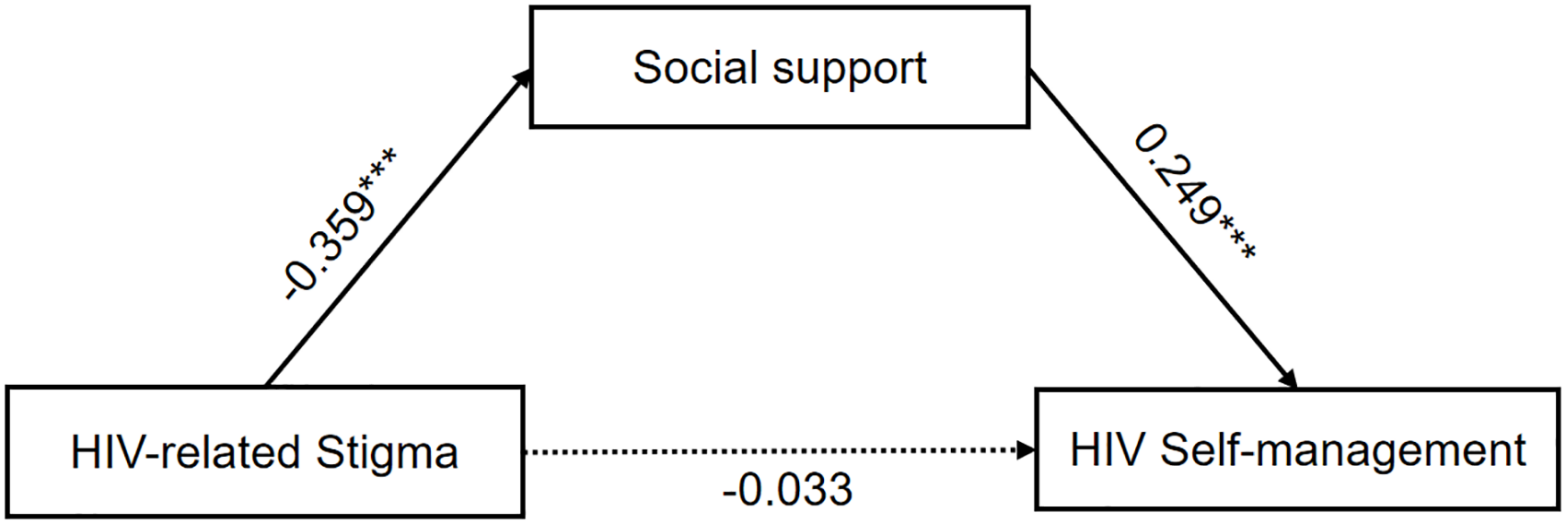
The single mediation role of social support in the relationship between HIV-related stigma and HIV self-management. The solid line and dashed line indicated significant and non-significant path coefficients, respectively. ^***^p < 0.001.

We examined the single mediation effect of social support and self-efficacy on the relationship between HIV-related stigma and HIV self-management after controlling comorbidities and drug side effects, respectively. In the model with social support as the mediator, lower HIV-related stigma predicts higher social support (*β* = −0.359, *p* < 0.001), and higher social support predicts better HIV self-management (*β* = 0.249, *p* < 0.001; Figure 2). A significant indirect effect of HIV-related stigma on HIV self-management *via* social support was found and the mediating effect value was −0.122 [Bootstrap 95% CI: −0.190, −0.064]. These results supported our hypothesis 1.

**Figure 3:**
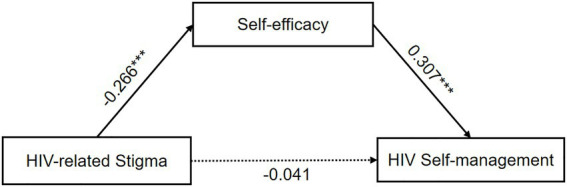
The single mediation role of self-efficacy in the relationship between HIV-related stigma and HIV self-management. The solid line and dashed line indicated significant and non-significant path coefficients, respectively. ^***^p < 0.001.

In the model with self-efficacy as the mediator, lower HIV-related stigma predicts higher self-efficacy (*β* = −0.266, *p* < 0.001), and higher self-efficacy predicts better HIV self-management (*β* = 0.307, *p* < 0.001; Figure 3). The path coefficients of HIV-related stigma on HIV self-management revealed that the indirect effect *via* self-efficacy was statistically significant. The mediating effect of self-efficacy was −0.095 [Bootstrap 95% CI: −0.155, −0.029]. These results also supported our hypothesis 2. Meanwhile, since the indirect effect of social support (−0.122) was slightly higher than self-efficacy (−0.095), social support was a stronger mediator than self-efficacy.

### Chain mediation model

**Figure 4:**
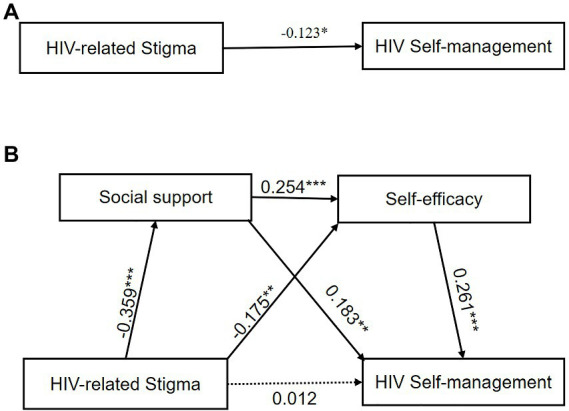
**(A)** The chain mediation role of social support in the relationship between HIV-related stigma and HIV self-management. **(B)** The solid line and dashed line indicated significant and non-significant path coefficients, respectively. ^*^p < 0.05, ^**^p < 0.01, ^***^p < 0.001.

Table 3 displays the coefficients and significance of each path in the chain mediation model. We found that the total effect (*β* = −0.123, *p* = 0.023; Figure 4A) of HIV-related stigma on HIV self-management was significant, which means that the higher HIV-related stigma, the less likely the HIV self-management among MSM living with HIV. The results confirmed that HIV-related stigma had a direct and significant negative prediction on the level of social support (*β* = −0.359, *p* < 0.001) and self-efficacy (*β* = −0.175, *p* = 0.001). Social support (*β* = 0.183, *p* = 0.001) and self-efficacy (*β* = 0.261, *p* < 0.001) directly and significantly predict the status of HIV self-management. Meanwhile, social support can also directly and significantly predict self-efficacy (*β* = 0.254, *p* < 0.001; Figure 4B).

**Table 3:** Regression coefficients in the serial mediation analysis.

Predictor variable	*R^2^*	*F*	*β*	*p*	*t*	*LLCI*	*ULCI*
Equation 1 Outcome variable: Social support							
Stigma	0.155	21.007	−0.359	<0.001	−7.063	−0.561	−0.317
Equation 2 Outcome variable: Self-efficacy							
Stigma	0.195	20.726	−0.175	0.001	−3.285	−0.493	−0.124
Social support			0.254	<0.001	4.815	0.217	0.517
Equation 3 Outcome variable: HIV self-management							
Stigma	0.158	12.817	0.012	0.826	0.220	−0.132	0.165
Social support			0.183	0.001	3.273	0.081	0.326
Self-efficacy			0.261	<0.001	4.723	0.118	0.286

Results of the chain mediation analyses in Table 4 indicated the total indirect effect was −0.135 [Bootstrap 95% CI: −0.191, −0.085], while the direct effect was 0.017 [Bootstrap 95% CI: −0.137, −0.0177], suggesting the direct effect was not significant. Specifically, the total indirect effect between the relations of HIV-related stigma on HIV self-management includes three pathways. The indirect mediating effect of HIV-related stigma on HIV self-management through social support and self-efficacy was −0.066 (*β* = 0.261, *p* < 0.001) and − 0.046 (*β* = 0.261, *p* < 0.001), respectively. The bootstrap’s 95% CI did not overlap the zero, indicating the two indirect pathways were statistically significant. Importantly, supporting our hypotheses, the indirect effect of chain mediation from HIV-related stigma to HIV self-management *via* social support and self-efficacy was significant, and the effect value was −0.024 [Bootstrap 95% CI: −0.042, −0.010]. In conclusion, the results mean that social support and self-efficacy play a full mediating role in the relationship between HIV-related stigma and HIV self-management. The chain mediation model is shown in Figure 4.

**Table 4:** Total, direct, and indirect effect of HIV-related stigma on HIV self-management though social support and self-efficacy.

Effect	Estimate	Boot SE	Bootstrap 95% CI	
			Low	High
Total effects	−0.168	0.072	−0.328	−0.046
Direct effects	0.017	0.080	−0.137	0.177
Total indirect effects	−0.135	0.027	−0.191	−0.085
X → M1 → Y	−0.066	0.023	−0.114	−0.024
X → M2 → Y	−0.046	0.018	−0.087	−0.014
X → M1 → M2 → Y	−0.024	0.008	−0.042	−0.010

X = HIV-related stigma; Y = HIV self-management; M1 = social support; and M2 = self-efficacy.

## Discussion

The present study explored the mediating role of social support and self-efficacy on the association between stigma and HIV self-management among MSM living with HIV based on the IFSMT. The results of parallel mediation analyses supported the hypotheses and confirmed the mediating role of social support and self-efficacy in the relationship between HIV-related stigma and HIV self-management. In addition, the chain mediation analyses suggested a significant serial between social support and self-efficacy.

In this study, the level of HIV self-management was higher than that found in general Ethiopian PLWH (Areri et al., 2020a), and lower than the finding reported among women living with HIV in the United States (Webel et al., 2013) and general PLWH in Korea (Kim et al., 2015). Compared to the United States and Korea, the implementation of HIV self-management model was later in china. We also found that patients living with comorbidities and side effects had a lower level of HIV self-management and controlled comorbidities and side effects as covariables in the mediating model. This finding was in accordance with the finding of Wang H. H. et al. (2019) and Areri et al. (2020b). This could be because comorbidities and side effects increase the complexity of medication management and pose a significant challenge to HIV self-management. Besides, those patients with comorbidities and drug side effects have worse health conditions and do not have enough energy to manage their disease (Areri et al., 2020b).

The current study also showed that higher HIV-related stigma was associated with a lower level of HIV self-management among MSM living with HIV after controlling comorbidities and drug side effects, which is obviously consistent with our first hypothesis and previous studies (Balaji et al., 2017; Turan et al., 2017; Xie et al., 2017). For instance, Balaji conducted a research among 9,819 MSM living with HIV indicating that a significant proportion of patients reported experiencing stigma and these experiences related to a host of risky sexual behaviors (Balaji et al., 2017). Xie also suggested that the family’s acceptance of the HIV diagnosis and absence of stigma had particularly positive influences on patients’ treatment adherence and emotional states (Xie et al., 2017). Additionally, as a sexual minority population, MSM living with HIV may experience intersectional forms of stigma for multiple stigmatized identities (e.g., sexual minority and HIV infection; Berger, 2022). Although we did not examine sexual minority stigma, previous studies have shown that multiple forms of stigma could interact to affect health behaviors among MSM living with HIV, especially in China, which emphasizes heterosexual marriage, having children, and filial piety to parents (Sun et al., 2020; Yang et al., 2020). Future studies may consider helping MSM living with HIV seek more sources to address intersectional stigma.

In line with our second expectations, the present study’s finding suggested that HIV-related stigma could work on HIV self-management *via* social support. A previous study conducted by Chan et al. (2020), suggested that psychosocial syndemics, including social isolation and poor mental health, mediated the relationship between HIV-related stigma and sexual risk behaviors. The support PLWH got from social networking, especially from families was seen to be helpful through reminding medication time, and providing information, material, financial, and mental support. Patients with more social support tend to adopt various strategies to achieve long-term HIV self-management goals and alleviate negative influences in daily life. However, those who experienced stigma often developed self-stigma toward their identity and were more likely to withdraw from social interaction due to fear of being stigmatized, excluded, or abandoned by family members and health providers, which in turn leads to limited use of social resources and poor health behaviors (Li et al., 2017; Turan et al., 2017; Schrimshaw et al., 2018). Areri et al. (2020b) proved that stigma was the main obstacles to share experiences with others to get access to health resources and support. Therefore, working to eliminate HIV-related stigma and establish a complete social support system are essential aspects of HIV self-management.

Consistent with the third hypothesis, decreasing HIV-related stigma may lead to a higher level of self-efficacy, which can positively impact behaviors. Our finding is congruent with a published study indicating that individuals with less internalized stigma were tend to be with more self-efficacy in adhering to ART, which facilitates them in conducting better HIV self-management regarding taking medicines (Yigit et al., 2020). HIV-related stigma increases the complexities surrounding HIV self-management, which may cause patients to perceive themselves as inferior and leads to less confidence in their abilities to overcome self-management tasks and achieve goals. From the perspective of the social cognitive theory (Bandura, 1989), self-efficacy could directly affect the formation of health behavioral motivation and control of HIV self-management. The present study further supports this finding and suggests that MSM living with HIV with a lower level of HIV-related stigma are more likely to sustain efforts in the face of frustrations and difficulties during the disease condition, which leads to changed HIV self-management.

Furthermore, our most notable finding is that HIV-related stigma had an impact on HIV self-management through the chain mediation of social support and self-efficacy, namely individuals with less stigma would firstly perceive more social support and then increase self-efficacy, which promotes their HIV self-management finally. This result supports our hypotheses and expands upon existing researches indicating that social support provides the necessary tools and information to assist patients in developing the skills to increase their confidence and regularly contact the healthcare system to address problems (Turan et al., 2016; Thaker et al., 2018). Cabral et al. (2018) confirmed that peer support could share common beliefs, help patients more effectively cope with stressful life events, and keep them engaged in the clinic. Voisin et al. (2017) also reported that MSM living with HIV with higher self-efficacy and an external environment of high social support were more likely to engage in health care. The IFSMT (Ryan and Sawin, 2009) offered a good explanation for such effects. This theory demonstrates that individual perceptions of external social resources, including perceived social support, affect patients’ desire and confidence to manage complex regiments, such as self-efficacy, thereby affecting engagement in positive and effective behaviors, which was manifested as HIV self-management in this study (Ryan and Sawin, 2009). Above all, extra social support is required to reduce patients’ perception of HIV-related stigma and help them come to believe in their ability to master skills to overcome problems, thus promotes HIV self-management.

The findings of this study extend our understanding of the status and influencing factors of HIV self-management and reveal ways in which HIV self-management could be improved. This study might have several clinical implications. First, the present study pinpoints the need for interventions to reduce HIV-related stigma. Structural efforts to educate the uninfected population and health providers on the vulnerability experienced by PLWH may reduce social stigma to patients and facilitate their participation in HIV care (Kerr et al., 2022). Effective intervention and strategies are needed to address HIV-related stigma as a barrier to HIV self-management among MSM living with HIV. Second, a better social support environment should be created that will improve patients’ family network and community relationships, and further enhance their confidence in their ability to achieve control of their health. Finally, while several interventions solely targeted stigma, social support (Giordano et al., 2016), or self-efficacy (Areri H. A. et al., 2020) as methods of improving HIV self-management, our study suggests that future interventions should combine the efforts in decreasing stigma, motivating social support and self-efficacy, and improving engagement of health care behaviors. Comprehensive interventions addressing HIV-related stigma, social support, and self-efficacy would generate more significant benefits for adjusting behaviors than that intervention targeting a single factor.

Inevitably, several limitations in this study should be acknowledged. First, the current study was a cross-sectional design, which excluded us from capturing longitudinal trends and establishing causality. Future longitudinal studies should be designed to explore causal inferences and further test and verify the reliability of our results (Zeng et al., 2020). Second, the sample only included MSM living with HIV from one city in China, so the generalizability of our study was limited and we cannot claim our findings fully represent the entire MSM living with HIV. Finally, our data were collected through self-report questionnaires or face-to-face interviews of patients with limited reading ability, which would be influenced by potential reporter bias due to social desirability (Yigit et al., 2020). Collecting data from other resources (e.g., family, friends, and healthcare) may minimize the influence of reporter bias. Moreover, future studies should use more objective measurements to collect data.

## Conclusion

The current study not only explored the mechanism of HIV-related stigma on HIV self-management in a Chinese MSM living with HIV, but also extended the application of IFSMT in the HIV self-management field. These findings indicated that social support and self-efficacy play as both individual mediators and chain mediators in the relationship. Given the mediating effect of social support and self-efficacy on HIV self-management, programs related to social support and self-efficacy should be designed to enhance HIV self-management among MSM living with HIV who report high levels of HIV-related stigma in China and other global settings.

## Data availability statement

The raw data supporting the conclusions of this article will be made available by the authors, without undue reservation.

## Ethics statement

This study involving human participants were reviewed and approved by Xiangya Nursing School, Central South University. The patients/participants provided their informed consent to participant in this study.

## Author contributions

YT, XX, and HW designed the work. YT and XX collected the data. YT, JM, and HW analyzed and drafted the manuscript. YT, XX, JM, and HW revised the manuscript. All authors contributed to the article and approved the submitted version.

## Funding

This work was supported by the Provincial Natural Science Foundation of Hunan Grant (2022JJ30769).

## Conflict of interest

The authors declare that the research was conducted in the absence of any commercial or financial relationships that could be construed as a potential conflict of interest.

## Publisher’s note

All claims expressed in this article are solely those of the authors and do not necessarily represent those of their affiliated organizations, or those of the publisher, the editors and the reviewers. Any product that may be evaluated in this article, or claim that may be made by its manufacturer, is not guaranteed or endorsed by the publisher.
